# 3-(4-Chloro­phenyl­sulfin­yl)-2,5-dimethyl-1-benzofuran

**DOI:** 10.1107/S1600536810035932

**Published:** 2010-09-11

**Authors:** Hong Dae Choi, Pil Ja Seo, Byeng Wha Son, Uk Lee

**Affiliations:** a Department of Chemistry, Dongeui University, San 24 Kaya-dong Busanjin-gu, Busan 614-714, Republic of Korea; b Department of Chemistry, Pukyong National University, 599-1 Daeyeon 3-dong, Nam-gu, Busan 608-737, Republic of Korea

## Abstract

In the crystal structure of the title compound, C_16_H_13_ClO_2_S, the 4-chloro­phenyl ring is oriented approximately perpendicular to the benzofuran ring plane [dihedral angle = 82.45 (5)°]. In the crystal, mol­ecules are linked by weak inter­molecular C—H⋯O and C—H⋯π inter­actions.

## Related literature

For the structures of related 3-(4-fluoro­phenyl­sulfin­yl)-2,5-dimethyl-1-benzofuran derivatives, see: Choi *et al.* (2010**a* ▶,b* ▶).
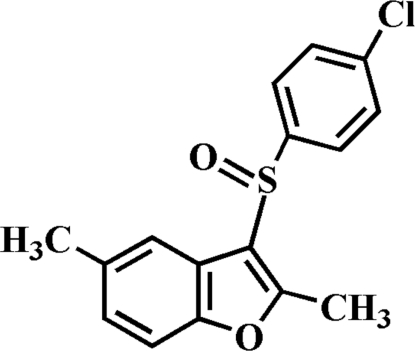


## Experimental

### 

#### Crystal data

C_16_H_13_ClO_2_S
*M* *_r_* = 304.77
Monoclinic, 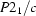

*a* = 12.7673 (19) Å
*b* = 11.0206 (18) Å
*c* = 11.1232 (17) Å
β = 113.674 (6)°
*V* = 1433.4 (4) Å^3^
*Z* = 4
Mo *K*α radiation
μ = 0.41 mm^−1^
*T* = 173 K
0.50 × 0.30 × 0.20 mm

#### Data collection

Bruker SMART APEXII CCD diffractometer
Absorption correction: multi-scan (*SADABS*; Bruker, 2009 ▶) *T*_min_ = 0.654, *T*_max_ = 0.746
12779 measured reflections
3542 independent reflections
3118 reflections with *I* > 2σ(*I*)
*R*_int_ = 0.029

#### Refinement

*R*[*F* ^2^ > 2σ(*F* ^2^)] = 0.036
*wR*(*F* ^2^) = 0.124
*S* = 1.03
3542 reflections
184 parameters
H-atom parameters constrained
Δρ_max_ = 0.31 e Å^−3^
Δρ_min_ = −0.31 e Å^−3^

### 

Data collection: *APEX2* (Bruker, 2009 ▶); cell refinement: *SAINT* (Bruker, 2009 ▶); data reduction: *SAINT*; program(s) used to solve structure: *SHELXS97* (Sheldrick, 2008 ▶); program(s) used to refine structure: *SHELXL97* (Sheldrick, 2008 ▶); molecular graphics: *ORTEP-3* (Farrugia, 1997 ▶) and *DIAMOND* (Brandenburg, 1998 ▶); software used to prepare material for publication: *SHELXL97*.

## Supplementary Material

Crystal structure: contains datablocks global, I. DOI: 10.1107/S1600536810035932/nc2196sup1.cif

Structure factors: contains datablocks I. DOI: 10.1107/S1600536810035932/nc2196Isup2.hkl

Additional supplementary materials:  crystallographic information; 3D view; checkCIF report

## Figures and Tables

**Table 1:** Hydrogen-bond geometry (Å, °) *Cg*1 is the centroid of the C1, C2, C7, O1, C8 furan ring.

*D*—H⋯*A*	*D*—H	H⋯*A*	*D*⋯*A*	*D*—H⋯*A*
C10—H10*A*⋯O2^i^	0.96	2.51	3.366 (2)	148
C15—H15⋯O2^ii^	0.93	2.60	3.353 (2)	139
C13—H13⋯*Cg*1^iii^	0.93	2.85	3.566 (2)	135

Symmetry codes: (i) 
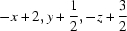
; (ii) 
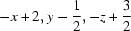
; (iii) 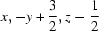.

**Fig. 1.**
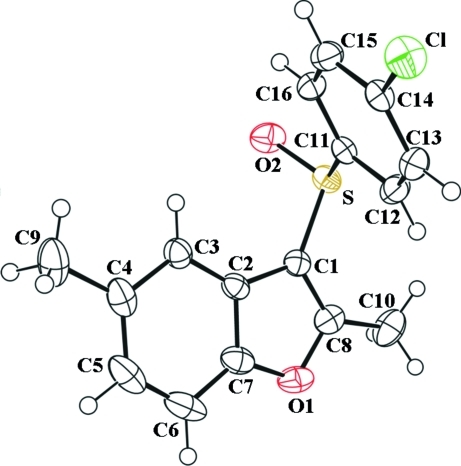
The molecular structure of the title compound with the atom numbering scheme. Displacement ellipsoids are drawn at the 50% probability level and H atoms are presented as a small spheres of arbitrary radius.

**Fig. 2.**
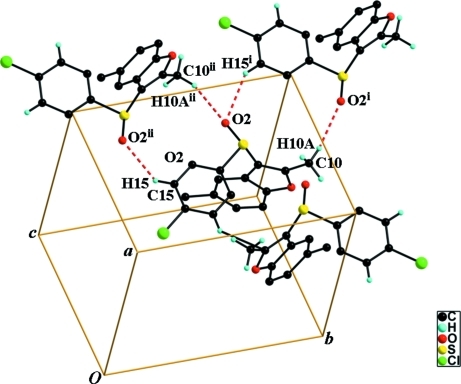
C—H···O interactions (dotted lines) in the crystal structure of the title compound. [Symmetry codes: (i) - x + 2, y + 1/2, - z + 3/2; (ii) - x + 2, y -1/2, - z +3/2.]

## supplementary crystallographic information

### Comment

As a part of our study on the substituent effect on the solid state structures of 3-(4-fluorophenylsulfinyl)-2,5-dimethyl-1-benzofuran analogues (Choi et al., 2010a, b/), we report the crystal structure of the title compound (Fig. 1).

The benzofuran unit is essentially planar, with a mean deviation of 0.004 (2) Å from the least-squares plane defined by the nine constituent atoms. The 4-chlorophenyl ring is nearly perpendicular to the benzofuran plane with a dihedral angle of 82.45 (5)°. In the crystal structure weak intermolecular C—H···O hydrogen bonding and C—H···π interaction are found (Fig. 2 and Table 1).

### Experimental

77% 3-chloroperoxybenzoic acid (269 mg, 1.2 mmol) was added in small portions to a stirred solution of 3-(4-chlorophenylsulfanyl)-2,5-dimethyl-1-benzofuran (317 mg, 1.1 mmol) in dichloromethane (40 mL) at 273 K. After being stirred at room temperature for 4h, the mixture was washed with saturated sodium bicarbonate solution and the organic layer was separated, dried over magnesium sulfate, filtered and concentrated at reduced pressure. The residue was purified by column chromatography (hexane–ethyl acetate, 1:1 v/v) to afford the title compound as a colorless solid [yield 77%, m.p. 440–441 K; R/_f_ = 0.71 (hexane–ethyl acetate, 1:1 v/v)]. Single crystals suitable for X-ray diffraction were prepared by slow evaporation of the solvent from a solution of the title compound in ethyl acetate at room temperature.

### Refinement

All H atoms were positioned geometrically and refined using a riding model, with  C—H = 0.93 Å for aryl, 0.97 Å for methyl H atoms. *U*_iso_(H) = 1.2*U*_eq_(C)for aryl and 1.5*U*_eq_(C) for methyl H atoms.

### Crystal data

**Table:**

C_16_H_13_ClO_2_S	*F*(000) = 632
*M**_r_* = 304.77	*D*_x_ = 1.412 Mg m^−^^3^
Monoclinic, *P*2_1_/*c*	Mo *K*α radiation, λ = 0.71073 Å
Hall symbol: -P 2ybc	Cell parameters from 8210 reflections
*a* = 12.7673 (19) Å	θ = 2.5–28.3°
*b* = 11.0206 (18) Å	µ = 0.41 mm^−^^1^
*c* = 11.1232 (17) Å	*T* = 173 K
β = 113.674 (6)°	Block, colourless
*V* = 1433.4 (4) Å^3^	0.50 × 0.30 × 0.20 mm
*Z* = 4	

### Data collection

**Table:**

Bruker SMART APEXII CCD diffractometer	3542 independent reflections
Radiation source: rotating anode	3118 reflections with *I* > 2σ(*I*)
graphite multilayer	*R*_int_ = 0.029
Detector resolution: 10.0 pixels mm^-1^	θ_max_ = 28.4°, θ_min_ = 1.7°
φ and ω scans	*h* = −17→16
Absorption correction: multi-scan (*SADABS*; Bruker, 2009)	*k* = −14→14
*T*_min_ = 0.654, *T*_max_ = 0.746	*l* = −14→13
12779 measured reflections	

### Refinement

**Table:**

Refinement on *F*^2^	Secondary atom site location: difference Fourier map
Least-squares matrix: full	Hydrogen site location: difference Fourier map
*R*[*F*^2^ > 2σ(*F*^2^)] = 0.036	H-atom parameters constrained
*wR*(*F*^2^) = 0.124	*w* = 1/[σ^2^(*F*_o_^2^) + (0.0772*P*)^2^ + 0.5141*P*] where *P* = (*F*_o_^2^ + 2*F*_c_^2^)/3
*S* = 1.03	(Δ/σ)_max_ = 0.001
3542 reflections	Δρ_max_ = 0.31 e Å^−^^3^
184 parameters	Δρ_min_ = −0.31 e Å^−^^3^
0 restraints	Extinction correction: *SHELXL97* (Sheldrick, 2008), Fc^*^=kFc[1+0.001xFc^2^λ^3^/sin(2θ)]^-1/4^
Primary atom site location: structure-invariant direct methods	Extinction coefficient: 0.014 (2)

### Special details

**Table:**

Geometry. All esds (except the esd in the dihedral angle between two l.s. planes) are estimated using the full covariance matrix. The cell esds are taken into account individually in the estimation of esds in distances, angles and torsion angles; correlations between esds in cell parameters are only used when they are defined by crystal symmetry. An approximate (isotropic) treatment of cell esds is used for estimating esds involving l.s. planes.
Refinement. Refinement of F^2^ against ALL reflections. The weighted R-factor wR and goodness of fit S are based on F^2^, conventional R-factors R are based on F, with F set to zero for negative F^2^. The threshold expression of F^2^ > 2sigma(F^2^) is used only for calculating R-factors(gt) etc. and is not relevant to the choice of reflections for refinement. R-factors based on F^2^ are statistically about twice as large as those based on F, and R- factors based on ALL data will be even larger.

### Fractional atomic coordinates and isotropic or equivalent isotropic displacement parameters (Å^2^)

**Table:**

	*x*	*y*	*z*	*U*_iso_*/*U*_eq_	
Cl	0.76980 (4)	0.23428 (4)	0.27031 (4)	0.04162 (15)	
S	0.92673 (3)	0.68708 (4)	0.64073 (4)	0.02874 (14)	
O1	0.66438 (10)	0.90669 (11)	0.50482 (12)	0.0371 (3)	
O2	0.95961 (10)	0.64186 (13)	0.77732 (11)	0.0398 (3)	
C1	0.79481 (13)	0.76039 (14)	0.59101 (14)	0.0261 (3)	
C2	0.69064 (12)	0.71954 (15)	0.60000 (14)	0.0264 (3)	
C3	0.65623 (13)	0.61696 (16)	0.64794 (15)	0.0303 (3)	
H3	0.7071	0.5536	0.6850	0.036*	
C4	0.54444 (14)	0.61099 (19)	0.63938 (17)	0.0386 (4)	
C5	0.47007 (15)	0.7081 (2)	0.5827 (2)	0.0485 (5)	
H5	0.3956	0.7033	0.5772	0.058*	
C6	0.50216 (16)	0.8100 (2)	0.5347 (2)	0.0477 (5)	
H6	0.4514	0.8734	0.4970	0.057*	
C7	0.61371 (14)	0.81344 (16)	0.54552 (16)	0.0333 (4)	
C8	0.77512 (14)	0.87155 (15)	0.53477 (15)	0.0315 (3)	
C9	0.50414 (17)	0.5015 (2)	0.6895 (2)	0.0520 (5)	
H9A	0.5632	0.4410	0.7170	0.078*	
H9B	0.4368	0.4692	0.6209	0.078*	
H9C	0.4867	0.5245	0.7627	0.078*	
C10	0.84732 (19)	0.95889 (18)	0.50136 (19)	0.0452 (4)	
H10A	0.8743	1.0205	0.5679	0.068*	
H10B	0.8031	0.9958	0.4181	0.068*	
H10C	0.9114	0.9170	0.4964	0.068*	
C11	0.87780 (11)	0.55732 (14)	0.53501 (14)	0.0253 (3)	
C12	0.82630 (14)	0.57394 (16)	0.40013 (15)	0.0319 (3)	
H12	0.8142	0.6517	0.3646	0.038*	
C13	0.79335 (14)	0.47361 (16)	0.31949 (15)	0.0324 (3)	
H13	0.7581	0.4831	0.2289	0.039*	
C14	0.81304 (12)	0.35892 (15)	0.37406 (15)	0.0272 (3)	
C16	0.89862 (12)	0.44303 (15)	0.58916 (15)	0.0293 (3)	
H16	0.9347	0.4336	0.6797	0.035*	
C15	0.86562 (14)	0.34154 (16)	0.50817 (16)	0.0314 (3)	
H15	0.8787	0.2637	0.5435	0.038*	

### Atomic displacement parameters (Å^2^)

**Table:**

	*U*^11^	*U*^22^	*U*^33^	*U*^12^	*U*^13^	*U*^23^
Cl	0.0453 (3)	0.0364 (3)	0.0423 (3)	−0.00681 (17)	0.0167 (2)	−0.01106 (18)
S	0.0214 (2)	0.0302 (2)	0.0333 (2)	−0.00229 (13)	0.00965 (15)	−0.00328 (15)
O1	0.0419 (6)	0.0266 (6)	0.0358 (6)	0.0078 (5)	0.0084 (5)	−0.0007 (5)
O2	0.0369 (6)	0.0435 (7)	0.0287 (6)	0.0039 (5)	0.0025 (5)	−0.0033 (5)
C1	0.0274 (7)	0.0247 (7)	0.0260 (7)	0.0000 (5)	0.0106 (5)	−0.0024 (6)
C2	0.0236 (6)	0.0305 (8)	0.0239 (6)	0.0025 (5)	0.0084 (5)	−0.0040 (6)
C3	0.0254 (7)	0.0368 (9)	0.0285 (7)	−0.0012 (6)	0.0106 (6)	−0.0007 (6)
C4	0.0296 (8)	0.0552 (12)	0.0337 (8)	−0.0072 (7)	0.0155 (6)	−0.0082 (8)
C5	0.0261 (8)	0.0683 (14)	0.0529 (11)	0.0017 (8)	0.0177 (8)	−0.0129 (10)
C6	0.0315 (8)	0.0547 (12)	0.0513 (11)	0.0167 (8)	0.0106 (8)	−0.0075 (9)
C7	0.0323 (8)	0.0319 (9)	0.0326 (8)	0.0053 (6)	0.0097 (6)	−0.0066 (6)
C8	0.0389 (8)	0.0262 (8)	0.0264 (7)	−0.0010 (6)	0.0099 (6)	−0.0048 (6)
C9	0.0408 (10)	0.0737 (15)	0.0464 (10)	−0.0196 (10)	0.0226 (8)	−0.0039 (10)
C10	0.0619 (12)	0.0323 (10)	0.0402 (9)	−0.0122 (8)	0.0193 (8)	0.0001 (8)
C11	0.0206 (6)	0.0290 (7)	0.0289 (7)	0.0005 (5)	0.0128 (5)	−0.0009 (6)
C12	0.0385 (8)	0.0286 (8)	0.0291 (7)	0.0031 (6)	0.0141 (6)	0.0056 (6)
C13	0.0361 (8)	0.0362 (9)	0.0252 (7)	0.0021 (6)	0.0125 (6)	0.0028 (6)
C14	0.0238 (6)	0.0300 (8)	0.0300 (7)	−0.0009 (5)	0.0132 (6)	−0.0027 (6)
C16	0.0285 (7)	0.0326 (8)	0.0257 (7)	0.0035 (6)	0.0097 (6)	0.0048 (6)
C15	0.0325 (7)	0.0276 (8)	0.0331 (8)	0.0020 (6)	0.0122 (6)	0.0035 (6)

### Geometric parameters (Å, °)

**Table:**

Cl—C14	1.7355 (16)	C8—C10	1.480 (2)
S—O2	1.4902 (13)	C9—H9A	0.9600
S—C1	1.7457 (15)	C9—H9B	0.9600
S—C11	1.7966 (16)	C9—H9C	0.9600
O1—C8	1.372 (2)	C10—H10A	0.9600
O1—C7	1.384 (2)	C10—H10B	0.9600
C1—C8	1.352 (2)	C10—H10C	0.9600
C1—C2	1.445 (2)	C11—C16	1.375 (2)
C2—C7	1.387 (2)	C11—C12	1.387 (2)
C2—C3	1.394 (2)	C12—C13	1.379 (2)
C3—C4	1.393 (2)	C12—H12	0.9300
C3—H3	0.9300	C13—C14	1.381 (2)
C4—C5	1.401 (3)	C13—H13	0.9300
C4—C9	1.504 (3)	C14—C15	1.381 (2)
C5—C6	1.375 (3)	C16—C15	1.391 (2)
C5—H5	0.9300	C16—H16	0.9300
C6—C7	1.381 (3)	C15—H15	0.9300
C6—H6	0.9300		
			
O2—S—C1	108.59 (7)	C4—C9—H9B	109.5
O2—S—C11	106.42 (8)	H9A—C9—H9B	109.5
C1—S—C11	97.08 (7)	C4—C9—H9C	109.5
C8—O1—C7	106.41 (13)	H9A—C9—H9C	109.5
C8—C1—C2	107.99 (14)	H9B—C9—H9C	109.5
C8—C1—S	122.84 (12)	C8—C10—H10A	109.5
C2—C1—S	129.17 (12)	C8—C10—H10B	109.5
C7—C2—C3	119.70 (14)	H10A—C10—H10B	109.5
C7—C2—C1	104.31 (14)	C8—C10—H10C	109.5
C3—C2—C1	135.99 (14)	H10A—C10—H10C	109.5
C4—C3—C2	118.93 (16)	H10B—C10—H10C	109.5
C4—C3—H3	120.5	C16—C11—C12	121.26 (15)
C2—C3—H3	120.5	C16—C11—S	119.15 (11)
C3—C4—C5	119.02 (18)	C12—C11—S	119.47 (12)
C3—C4—C9	120.41 (18)	C13—C12—C11	119.08 (15)
C5—C4—C9	120.57 (17)	C13—C12—H12	120.5
C6—C5—C4	123.03 (17)	C11—C12—H12	120.5
C6—C5—H5	118.5	C12—C13—C14	119.60 (14)
C4—C5—H5	118.5	C12—C13—H13	120.2
C5—C6—C7	116.50 (17)	C14—C13—H13	120.2
C5—C6—H6	121.8	C13—C14—C15	121.69 (15)
C7—C6—H6	121.8	C13—C14—Cl	118.62 (12)
C6—C7—O1	126.38 (17)	C15—C14—Cl	119.68 (13)
C6—C7—C2	122.83 (18)	C11—C16—C15	119.85 (14)
O1—C7—C2	110.79 (14)	C11—C16—H16	120.1
C1—C8—O1	110.50 (14)	C15—C16—H16	120.1
C1—C8—C10	133.36 (16)	C14—C15—C16	118.51 (15)
O1—C8—C10	116.14 (16)	C14—C15—H15	120.7
C4—C9—H9A	109.5	C16—C15—H15	120.7
			
O2—S—C1—C8	131.30 (14)	C1—C2—C7—O1	0.08 (17)
C11—S—C1—C8	−118.68 (14)	C2—C1—C8—O1	−0.52 (17)
O2—S—C1—C2	−48.63 (16)	S—C1—C8—O1	179.54 (10)
C11—S—C1—C2	61.39 (15)	C2—C1—C8—C10	179.19 (17)
C8—C1—C2—C7	0.26 (17)	S—C1—C8—C10	−0.7 (3)
S—C1—C2—C7	−179.80 (12)	C7—O1—C8—C1	0.56 (17)
C8—C1—C2—C3	−179.12 (17)	C7—O1—C8—C10	−179.21 (14)
S—C1—C2—C3	0.8 (3)	O2—S—C11—C16	−13.00 (13)
C7—C2—C3—C4	0.3 (2)	C1—S—C11—C16	−124.81 (12)
C1—C2—C3—C4	179.60 (16)	O2—S—C11—C12	171.00 (12)
C2—C3—C4—C5	0.1 (2)	C1—S—C11—C12	59.20 (13)
C2—C3—C4—C9	179.73 (16)	C16—C11—C12—C13	1.4 (2)
C3—C4—C5—C6	−0.1 (3)	S—C11—C12—C13	177.30 (12)
C9—C4—C5—C6	−179.70 (19)	C11—C12—C13—C14	−0.6 (2)
C4—C5—C6—C7	−0.3 (3)	C12—C13—C14—C15	−0.3 (2)
C5—C6—C7—O1	−179.62 (17)	C12—C13—C14—Cl	179.56 (12)
C5—C6—C7—C2	0.8 (3)	C12—C11—C16—C15	−1.3 (2)
C8—O1—C7—C6	179.97 (17)	S—C11—C16—C15	−177.26 (12)
C8—O1—C7—C2	−0.38 (17)	C13—C14—C15—C16	0.3 (2)
C3—C2—C7—C6	−0.7 (2)	Cl—C14—C15—C16	−179.50 (11)
C1—C2—C7—C6	179.74 (16)	C11—C16—C15—C14	0.5 (2)
C3—C2—C7—O1	179.58 (13)		

### Hydrogen-bond geometry (Å, °)

**Table:**

Cg1 is the centroid of the C1, C2, C7, O1, C8 furan ring.

**Table:**

*D*—H···*A*	*D*—H	H···*A*	*D*···*A*	*D*—H···*A*
C10—H10A···O2^i^	0.96	2.51	3.366 (2)	148
C15—H15···O2^ii^	0.93	2.60	3.353 (2)	139
C13—H13···Cg1^iii^	0.93	2.85	3.566 (2)	135

Symmetry codes: (i) −*x*+2, *y*+1/2, −*z*+3/2; (ii) −*x*+2, *y*−1/2, −*z*+3/2; (iii) *x*, −*y*+3/2, *z*−1/2.
